# Clinico-Immunological Effects of a Single-Agent CDK4/6 Inhibitor in Advanced HR+/HER2− Breast Cancer Based on a Window of Opportunity Study

**DOI:** 10.3390/cimb44090292

**Published:** 2022-09-15

**Authors:** Alberto D’Angelo, Fabiola Giudici, Robert Chapman, Jacob Darlow, Huseyin Kilili, Navid Sobhani, Mattia Cinelli, Maria Rosa Cappelletti, Carla Strina, Manuela Milani, Daniele Generali

**Affiliations:** 1 Department of Biology and Biochemistry, University of Bath, Bath BA2 7 AY, UK; 2 Department of Biostatistics and Epidemiology, Gustave Roussy, Paris-Saclay University, 91190 Gif-sur-Yvette, France; 3 Department of Medicine, The Princess Alexandra Hospital, Harlow CM20 1 QX, UK; 4 Milner Centre for Evolution, Department of Biology and Biochemistry, University of Bath, Bath BA2 7 AY, UK; 5 Section of Epidemiology and Population Science, Department of Medicine, Baylor College of Medicine, Houston, TX 77030, USA; 6 UOC Multidisciplinare di Patologia Mammaria e Ricerca Traslazionale, Azienda Socio-Sanitaria Territoriale di Cremona, 126100 Cremona, Italy; 7 Dipartimento Universitario Clinico di Scienze Mediche, Chirurgiche e della Salute, Università degli Studi di Trieste, 34129 Trieste, Italy

**Keywords:** breast cancer, metastasis, CDK4/6 inhibitors, immunomodulation

## Abstract

**Background**: Cyclin-dependent kinase 4/6 inhibitors (CDK4/6 i), abemaciclib, palbociclib, and ribociclib, have been FDA-approved for the treatment of hormone receptor-positive (HR+), HER2−negative (HER2−) advanced breast cancer (aBC). This targeted therapy has revived hope in those aBC patients who did not respond to standard therapies. Interestingly, when administered as a single agent, CDK4/6 modulated several peripheral blood cells after a short-course treatment of 28 days. However, the impact of these immune effects has yet to be thoroughly investigated. **Methods**: We administered abemaciclib, palbociclib, and ribociclib monotherapy to 23 patients with HR+/HER2− metastatic breast cancer. The aim is to investigate the impact of on-treatment modifications on peripheral blood cells and their composite scores in patients after a 28-day course of CDK4/6 i alone. **Results**: In the current study, we observed a significant decrease in neutrophils (*p*-value < 0.001) for patients treated with abemaciclib, palbociclib, and ribociclib. An overall decrease of T_regs_ was observed and potentially linked to palbociclib treatment. The neutrophile to lymphocyte (N/L) ratio was also decreased overall and potentially linked to abemaciclib and palbociclib treatment. Platelets were decreased in patients administered with abemaciclib. Notably, the radiometabolic response was available only for those patients treated with ribociclib and abemaciclib, and only those lesions treated with ribociclib reached statistical relevance. **Conclusions**: Our study strongly supports the notion that CDK4/6 inhibitors induce tumour immune modulation. N/L ratio and platelet levels decreased due to treatment. Future studies should test whether patients would benefit from immunomodulators in association with CDK4/6 agents in a larger clinical trial. Moreover, the CDK4/6-induced immune modulation could also be considered a potential predictive clinical factor in HR+/HER2− advanced breast cancer.

## 1. Introduction

Breast cancer (BC) is the most diagnosed cancer in women worldwide, with over 2.2 million new cases in 2021 [1]. Two out of three cases of BC include hormone receptor-positive (HR+) and human epidermal growth factor receptor 2-negative (HER2−) histology [2]. Endocrine therapy (ET) combined with cyclin-dependent kinase 4 and 6 (CDK4/6) inhibitors, namely, abemaciclib, palbociclib, and ribociclib, is currently the standard first-line hormonal palliative treatment for HR+/HER2− metastatic BC (mBC), both in pre-and postmenopausal patients [3].

CDK4/6 act to control cell cycle progression through the early G1 phase and are positively regulated by the interaction with cyclins (e.g., D1/D2/D3) and negatively regulated by tumour suppressors (e.g., p16 INK4 A) that stop CDK4/6 interaction with D-type cyclins [4]. Once the stimulatory signal is provided, cyclin D-CDK4/6 complexes hyper-phosphorylate retinoblastoma proteins (RB), releasing E2 F transcription factors, which guide the transcription of genes responsible for cell cycle progression. RB and p16 INK4 A play a pivotal role in the regulation of cell proliferation, and several inactivating mutations in R.B. and p16INK4A genes have been identified in various cancer types [4,5].

Broadly targeting the cell cycle using CDK inhibitors started in the early 1990s with dinaciclib and flavopiridol [6,7,8]. Unfortunately, the outcome of these preliminary trials was underwhelming, primarily due to poor patient selection criteria and low specificity of compounds. Joint efforts were made to develop more effective CDK4/6 inhibitors, which ultimately led to the development of palbociclib (PD0332991), an orally available CDK4/6 inhibitor synthesized by Pfizer [9]. In May 2015, palbociclib was granted breakthrough therapy designation and, subsequently, FDA approval for the treatment of postmenopausal, HR+/HER2− breast cancer patients in association with fulvestrant (a selective HR degrader) or an aromatase inhibitor. Further selective CDK4/6 inhibitors, including abemaciclib/LY2835219 (Eli-Lily, Indianapolis, IN, USA) and ribociclib/LEE011 (Novartis, Basel, Switzerland), were developed with additional properties and safer toxicity profiles.

Abemaciclib, palbociclib, and ribociclib demonstrated increased progression-free survival from 5 to 10 months in crucial clinical trials [10,11,12,13,14,15,16]. Furthermore, a recent meta-analysis showed that CDK4/6 inhibitors in association with hormonal therapy improved overall survival (OS) (independent of endocrine sensitivity, menopausal status, visceral involvement, and age) when compared to ET alone [17]. For the reasons above, CDK4/6 therapy associated with ET is considered the gold-standard treatment for HR+/HER2− mBC with no extensive visceral involvement. Despite the survival benefits of CDK4/6 inhibitors, resistance occurs in a large fraction of patients (approximately 15% and 30% in those administered with CDK4/6 plus anti-aromatase inhibitors or fulvestrant, respectively) [18]. Further research by Turner et al. showed that a potential resistance mechanism to CDK4/6 treatment—and thus leading to tumour relapse—might involve specific mutations occurring in *Rb*, *ERS1,* and *PIK3 CA* genes [19]. Artega et al. additionally observed that *FGFR1* gene amplifications can be a resistance mechanism to CDK4/6 inhibitors and result in tumour relapse [20]. Notably, there are currently no predictive factors of response to CDK4/6 inhibitors, and ER positivity is deemed to be the only robust marker in predicting CDK4/6 response in breast cancer patients.

However, BC progression is closely associated with the immune system [21]. Adaptive and innate immunity affect the occurrence, development, and spread of breast cancer [22]. Moreover, immune tolerance is one of the crucial factors in the development of tumour resistance to treatments. Available evidence suggests that tumour-infiltrating lymphocytes play an essential role in tumour monitoring and are related to increased survival [22]. Previous studies have mainly focused on the local immune response within the tumour microenvironment; however, several studies have shown that the local antitumour immune response is connected to the systemic immune response [23]. Additionally, some trials have observed that unexpected T cell clones in the peripheral blood might lead to better outcomes [18]. The role of circulating immune cells in the control of tumour response to therapies has gained widespread interest over the last decade, and baseline lymphopenia has been observed as a potential prognostic factor in different types of cancer [18].

However, very little has been published concerning the predictive and prognostic role of peripheral blood lymphocytes (PBLs). In our study, we focus on PBLs and platelets. These samples are easy to access, simple to analyse, allow for dynamic monitoring, and offer robust homogeneity compared to TILs. Only a few studies have observed that the neutrophil-to-lymphocyte ratio (NLR) and the platelet-to-lymphocyte ratio (PLR) before neoadjuvant treatment, mostly including taxanes and anthracyclines, are associated with responsiveness and prognosis [24,25,26].

The present study aimed to explore the biological effect of short abemaciclib, palbociclib, and ribociclib (28 days of continuous treatment for abemaciclib, 21 days of treatment and one week off for palbociclib and ribociclib) on circulating immune cells and platelets, in 23 advanced ER+/HER2− BC patients. Additionally, we investigated the radiometabolic effect of the aforementioned chemotherapeutic agents on 9 out of 23 patients (24 lesions in total). The data collected could provide a reference base for exploring other biomarkers to streamline treatment decisions in the future by enabling early identification of patients responding to therapy.

## 2. Materials and Methods

### 2.1. Study Design and Eligibility Criteria

From January 2019 to February 2020, 23 treatment-naïve patients diagnosed with metastatic Luminal A and B breast cancer (stage IV, according to the AJCC 8th edition) eligible for systemic therapy were enrolled at the ASST of Cremona Hospital. Only those patients with an Eastern Cooperative Oncology Group (ECOG) performance status of 1 or below, with good baseline cardiac, renal, hepatic, and haematological functions, were recruited for the study. The study was conducted following the Declaration of Helsinki, Good Clinical Practice principles, and all local regulations. The study (Mozart Trial EUDRACT 2018-002116-26) was granted ethical approval by the ethical committee of the ASST of Cremona Hospital (IRB Approval 27.12.18 n 433), and all patients involved provided written consent.

### 2.2. Study Objective and Endpoints

This study’s main objective was to investigate the biological effects of the CDK4/6 i administered alone in a window of opportunity fashion in metastatic BC patients [17,27]. The primary endpoint was the exploratory evaluation of early changes in circulating immune cells for the overall cohort of patients (n = 23) induced by each agent under investigation. Secondary endpoints included the experimental assessment of radiometabolic (PET/TC) response to either abemaciclib, palbociclib, or ribociclib short course.

### 2.3. Study Treatments and Procedures

Patients were assigned to orally receive either abemaciclib (n = 8, 34.8%), palbociclib (n = 6, 26.1%), or ribociclib (n = 9, 39.1%) at a dose of 150 mg/bid daily, 125 mg/daily, and 600 mg daily, respectively (28 days of continuous treatment for abemaciclib, 21 days of treatment and one week off for palbociclib and ribociclib) before starting with the standard combination with letrozole 2.5 mg/daily [17,27]. Blood sampling was performed at baseline and after 28 days of treatment. Treatment was stopped in cases of unacceptable toxicity or imaging confirmation of disease progression. Toxicities were classified according to the CTCAE version 4.03. All patients enrolled in the study experienced at least one adverse event (AE), and all except 1 patient in the 600 mg dose group experienced at least one grade 3 AE. The most frequent AEs were hematologic, followed by nausea and increased laboratory values. The most frequent AEs of any grade were hematologic, a finding consistent with other studies of CDK4/6 i. The most frequent treatment-related ≥3 grade AEs were leukopenia (82%), neutropenia (72%), and lymphopenia (69%). However, these AEs were generally mild and none of them individually occurred in more than two patients enrolled in the trial. All events resolved without clinical action. None of the patients discontinued treatment due to an AE.

FDG^18^-PET/CT scan was conducted at baseline and after 28 days of treatment ± 2 days. Patients were considered responsive to agents under investigation when a reduction of SUV_max_ was significant after 28 days of treatment. Conversely, patients were considered nonresponsive when stability or increase in SUV_max_ was observed according to PERCIST criteria. The same radiologist evaluated PET/CT responses at baseline and after 28 days of treatment.

### 2.4. Flow Cytometry Analysis

Flow cytometry analyses were used to investigate the circulating immune cells via whole blood samples, which were taken before and following either abemaciclib, palbociclib, or ribociclib administration. The use of Becton Dickinson (BD) FACSCanto^TM^ and BD FACSCanto II^TM^ with BD^TM^ Cytometer Setup and Tracking (CS&T) control enabled signals to be reproducible and comparable in spite of any fluctuation in environmental conditions. BDFACSC Diva Software was used for the acquisition and assessment of at least 1.5 × 10^6^. The BD Multitest 6-color TBNK kit (BD) was used for the assessment of the following subpopulations: lymphocytes B, natural killer (NK), and T cells with CD4 and CD8 subpopulations.

### 2.5. Statistical Analysis

The distribution of continuous variables was inspected for normality using the Shapiro–Wilk test. Data were reported using the mean and standard deviation (SD) if normally distributed or median and range (minimum–maximum) if not. Categorical variables were described by frequency and proportion. Parameters of interest, including SUV, the overall count of lymphocytes (CD45+), count of lymphocytes B (CD19+), NK (CD16+CD56+), T (CD3+), T Helper (CD3+CD4+), T Suppressor (CD3+CD8+), T_regs_ (CD3+CD4+CD25++CD127−), neutrophils, platelets, CD4/CD8, N/L (neutrophil to lymphocytes) and P/L (platelets to lymphocytes) ratios before and following 28-day treatment, were compared using the Student’s *t*-test and the Wilcoxon matched-pairs test for continuous data. We used waterfall plots to provide an overview of how patients responded to the therapies. Statistical analysis was achieved by R software (version 4.1.21, R Core Team (2020). R: A language and environment for statistical computing. R Foundation for Statistical Computing, Vienna, Austria, URL https://www.R-project.org/ (accessed on 1 February 2022). A *p*-value < 0.05 was deemed to be significant, although formal comparisons were only exploratory.

## 3. Results

### 3.1. Patients and Tumour Characteristics

A total of 23 patients were enrolled. The median patient age was 62.8, and surgery technique, namely, either conservative or mastectomy as initial treatment, was equally distributed. All patients were diagnosed with metastatic stage IV disease. In case of relapse, metastasis was biopsied for biomarker evaluation. In terms of molecular subtype, 16 (69.6%) patients carried the Luminal B subtype (ER-positive and/or PR-positive tumours with negative HER2 and low Ki67 proliferation (<14%)), while 7 (30.4%) patients carried the Luminal A subtype (ER-positive and/or PR-positive tumours with positive/negative HER2 and high Ki67 proliferation (≥14%)) [28]. All metastases were hormone-sensitive. Eight patients were administered with abemaciclib (34.8%), six patients received palbociclib (26.1%), and nine were administered with ribociclib (39.1%). At present, 14 patients (60.9%) are still alive, while 9 (39.1%) are deceased (median follow-up of 9.5 years). Patients’ characteristics are summarised in Table 1.

### 3.2. Overall Treatment Effect on Immune Circulating Cells and Platelets

**Figure 1:**
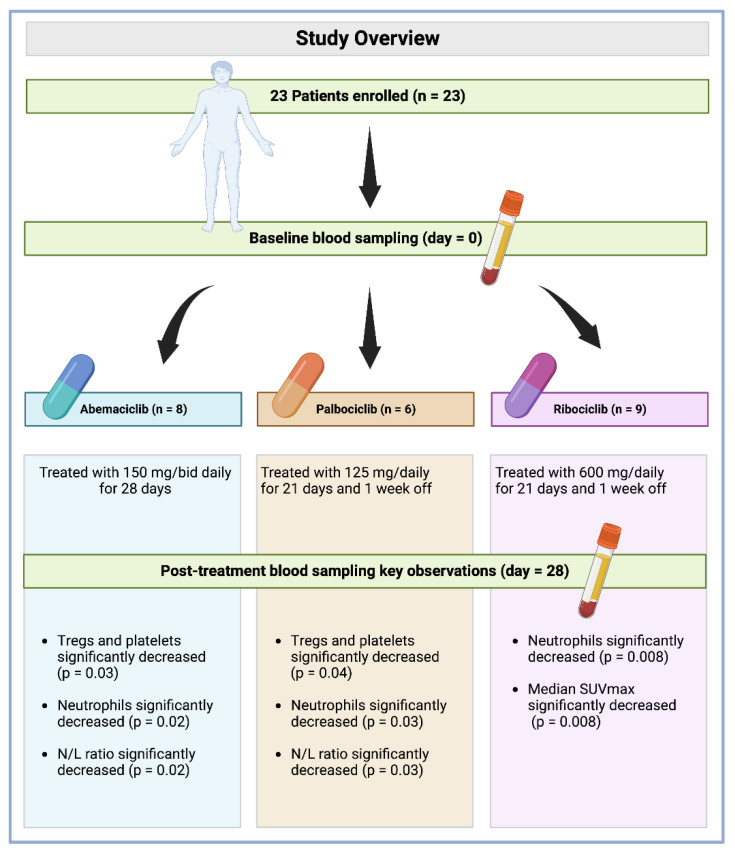
Study overview with key observation summary per drug.

Immune circulating subpopulations and platelets were evaluated at baseline and after 28 days of treatment (Figure 1). We observed a decrease in the number of platelets and all immune cell subpopulations, including the overall count of lymphocytes (CD45+) (from an average of 1664.7 cells/μL to 1552.4 cells/μL), lymphocytes B (CD19+) (from an average of 198 cells/μL to 125 cells/μL), NK cells (CD16+CD56+) (from an average of 311.6 cells/μL to 280.7 cells/μL), T cells (CD3+) (from an average of 1134.3 cells/μL to 1076.3 cells/μL), T Helper cells (CD3+CD4+) (from an average of 680.0 cells/μL to 654.2 cells/μL), T Suppressor cells (CD3+CD8+) (from an average of 470.7 cells/μL to 396.6 cells/μL), T_regs_ (CD3+CD4+CD25+CD127−) (from an average of 59 cells/μL to 46 cells/μL), neutrophils (from an average of 3.45 cells/μL to 1.33 cells/μL), platelets (from an average of 250.8 cells/μL to 228.7 cells/μL), CD4/CD8 ratio (from an average of 1.74 cells/μL to 1.56 cells/μL), and N/L ratio (neutrophil to lymphocytes) (from an average of 2.23 to 1.00). Contrastingly, the P/L ratio (platelets to lymphocytes) was increased (from an average of 167.6 cells/μL to 1784.2 cells/μL). Among these values, only those regarding T_regs_, neutrophils, and the N/L ratio were reported to be statistically significant, with *p*-values of 0.02, <0.001, and <0.001, respectively (Table 2).

### 3.3. Treatment Effect on Immune Circulating Cells and Platelets According to the Therapeutic Agent

**Figure 2:**
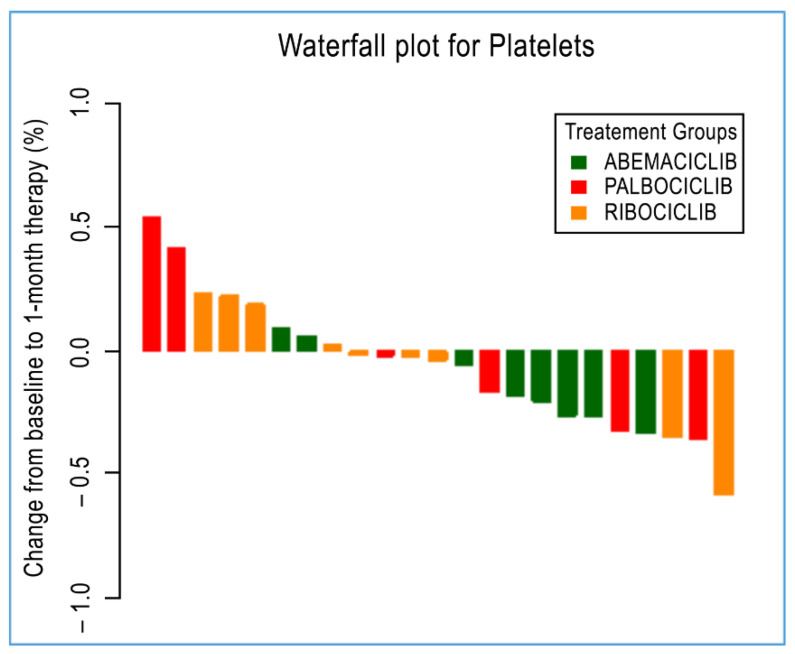
A waterfall plot of post-treatment changes in platelets from baseline for 23 patients.

Immune circulating subpopulations and platelets were further evaluated at baseline and after 28 days according to each specific treatment. In agreement with previously reported results, we observed an overall decrease—after 28-day therapy for each drug—in the number of T_regs_, neutrophils, platelets, and N/L ratio. However, T_regs_ and platelets were found to be decreased at a statistically significant level in those patients administered with palbociclib (from an average of 59 cells/μL to 44 cells/μL, *p* = 0.04) and abemaciclib (from an average of 224.4 cells/μL to 187.8 cells/μL, *p* = 0.03, Figure 2). Interestingly, neutrophils were decreased at a statistically significant level in patients administered with all treatments under evaluation, namely, abemaciclib (from an average of 3.32 cells/μL to 1.32 cells/μL, *p* = 0.02), palbociclib (from an average of 3.31 cells/μL to 1.46 cells/μL, *p* = 0.03), and ribociclib (from an average of 3.45 cells/μL to 1.39 cells/μL, *p* = 0.008). Additionally, the N/L ratio was also reported to be significantly decreased in those patients administered with abemaciclib (from an average of 2.07 cells/μL to 0.84 cells/μL, *p* = 0.02) and palbociclib (from an average of 2.48 cells/μL to 1.11 cells/μL, *p* = 0.03) (Table 3). No statistically significant difference was observed for other immune cells.

### 3.4. Radiometabolic Biomarkers According to Therapeutic Response

**Figure 3:**
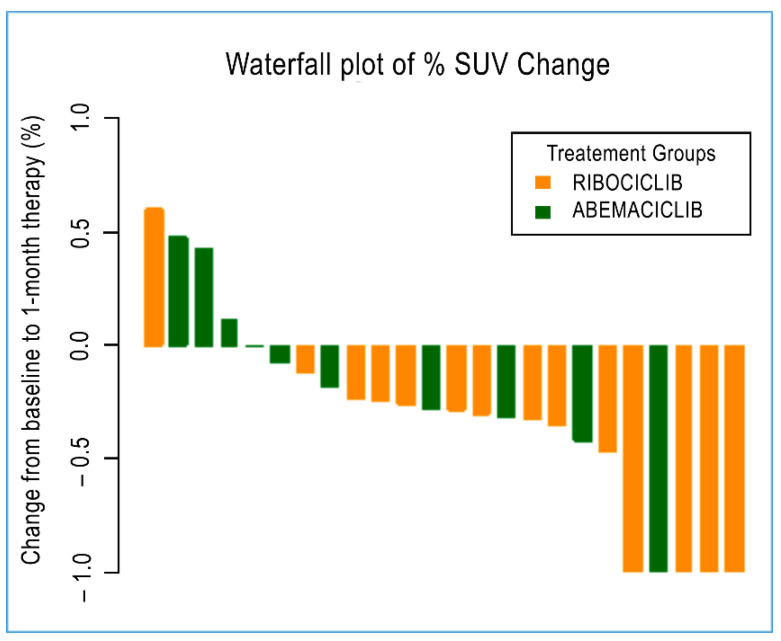
A waterfall plot of post-treatment changes in SUV from baseline for 24 evaluable lesions.

Radiometabolic response at baseline and after 28 days (28 days of continuous treatment for abemaciclib, 21 days of treatment and one week off for palbociclib and ribociclib) was available for only 9 out of 23 patients and a total of 24 malignant lesions. Four of these patients were administered with abemaciclib (10 malignant lesions), and five were issued with ribociclib (14 malignant lesions), as reported in Table 4. Overall, a significant decrease in median SUV_max_ was reported among all 24 lesions from baseline. The median SUV_max_ measure decreased from an average value of 4.8 at diagnosis to 2.9 at the end of treatment (*p* = 0.006). Regarding the specific treatment, only those lesions treated with ribociclib reached statistical significance (*p* = 0.008), with a decrease in median SUV_max_ from an average value of 4.75 at diagnosis to 2.9 at the end of treatment. Those lesions treated with abemaciclib, although showing a decreasing trend for SUV_max_, did not achieve statistical relevance (Figure 3).

## 4. Discussion

Despite the number of studies already published, no clear indication of a role for peripheral systemic immunity has been observed so far in patients administered with CDK4/6 i, partly due to the lack of standardized cut-off values and the limited number of patients enrolled.

In our study, we first confirmed that CDK4/6 i-based therapy resulted in a considerable decrease in peripheral blood white cells (primarily neutrophils), as previously observed in phase II and phase III studies investigating CDK4/6 i agents [15,29,30,31,32,33]. The overall decrease in total white blood cell and neutrophil counts is a well-established and common adverse event (AE) of palbociclib, ribociclib, and abemaciclib-based therapy [10,15,29,30,31,32]. This side effect is likely due to the CDK6 inhibition of transcription modulation of the EGR1 gene, which results in the impairment of hematopoietic stem cell proliferation and differentiation in bone marrow [34]. In a pooled safety analysis (including the PALOMA 1, 2, and 3 trials) 1% of patients reported grade 3–4 lymphopenia and the level of any grade 5-year lymphopenia was 2.8% [10,11,35]. In the MONALEESA 2 trial, grade 3 and 4 lymphopenia rates were 7% and 0.9%, respectively [12]. However, the overall impact of CDK4/6 i-based therapy on peripheral blood counts in PALOMA, MONARCH, and MONALEESA trials has not been accurately discussed, and only a single case study describes a patient who developed neutropenia, thrombocytopenia, and anaemia following a palbociclib regimen [36]. It is worth mentioning that lymphopenia is a robust predictor of chemotherapy-induced toxicity and is also a predictive factor of treatment efficacy in lung, breast, and colon cancer [37,38,39]. It has been observed that palbociclib and ribociclib have higher selectivity towards CDK4 and CDK6 than abemaciclib, although the latter demonstrated higher off-target activity and approximately 13 times more specificity for CDK4 than CDK6 [40,41]. For this reason, the incidence of neutropenia is more commonly observed in patients treated with abemaciclib. Unlike chemotherapy-induced neutropenia, CDK4/6 i-induced neutropenia is not correlated with an increased risk of developing severe infections, possibly because CDK4/6 i does not result in irreversible damage and apoptotic cell death in white blood cell precursors in the bone marrow [42]. On the contrary, CDK4/6 i can enable cytotoxic activity by mitigation of myeloid-cell-mediated immunosuppression [43]. In light of the above, our analysis is an emerging study describing the significant reduction of peripheral blood counts following single-agent abemaciclib, palbociclib, and ribociclib administration.

A decline in neutrophil-to-lymphocytes (N/L) ratio was also observed during this study, mainly in the abemaciclib and palbociclib groups. No statistically significant differences were observed for the ribociclib group. It was recently observed that peripheral immunity and inflammation balance indicators such as the N/L ratio might be a predictive response marker and correlate with good outcomes [24]. Vernieri et al. recently showed that the N/L ratio was associated with response to platinum agents in metastatic triple-negative breast cancer patients [44]. Further studies have investigated the N/L ratio in breast cancer patients following neoadjuvant chemotherapy [45,46,47]. Overall, a low N/L ratio potentially suggests a systemic background of decreased immune activation and resulting inflammation, leading to better treatment response [24].

A decrease in the overall count of peripheral regulatory T cells (T_regs_) was also observed. To the best of our knowledge, this is one of the first studies observing an in vivo correlation between CDK4/6 i administration and a decrease in peripheral T_regs_ in patients diagnosed with ER+/HER2− breast cancer. The ability of CDK4/6 i to decrease T_reg_ count has been described only in preclinical studies [48]. In this study, we observed a significant decrease in T_reg_ levels, particularly in patients administered palbociclib. Overall cohort data reported a reduction of T_regs_ cells, particularly the effector T_regs_ subset (CD3+CD4+CD25++CD127−), which is involved in immunosuppressive functions.

Ultimately, we would conclude that ribociclib showed the most significant effect on tumour shrinkage—according to the radiometabolic response, whilst no statistically significant reduction was reached in those lesions treated with abemaciclib. We cannot exclude that tumour shrinkage induced by abemaciclib treatment did not reach statistical relevance due to the limited number of lesions under investigation. However, abemaciclib appeared to be the CDK4/6 with the strongest effect, according to its ability to reduce neutrophil, N/L ratio, and platelet levels; this is likely due to its higher selectivity for CDK4 and CDK9 [49].

With regards to thrombocytopenia, although we observed decreased platelet counts among the entire cohort of patients, this was most apparent in patients treated with abemaciclib. Thrombocytopenia is another emerging side effect of CDK4/6 i treatment reported in PALOMA−2, PALOMA−3, MONALEESA, and MONARCH−3 trials. More specifically, any-grade thrombocytopenia was observed in 15–25% of patients in PALOMA−2 and PALOMA−3 trials, with grade 3 and grade 4 thrombocytopenia observed in 1 to 2% and 0.2 to 1% of patients, respectively [29,30]. The incidence of any-grade thrombocytopenia was 9.3 to 11% in MONALEESA trials, with grade 3 and grade 4 thrombocytopenia observed in 1% of patients [33,50]. As for abemaciclib, any grade thrombocytopenia occurred in 36.2 to 53.2% of patients in MONARCH−2 and MONARCH−3 trials, with grade 3 and grade 4 thrombocytopenia reported in 1.3 to 2% and 0.6 to 1.4% of patients, respectively [13,15].

## 5. Conclusions

Collectively, these data strongly support the idea that CDK4/6 inhibitors induce tumour immune modulation. Moreover, when considering the critical need for the identification of patients with either a good treatment response or resistance to CDK4/6 agents, CDK4/6 immune modulation should be evaluated as a novel and robust predictive factor. Furthermore, modifications of peripheral immune subsets and tumour-infiltrating lymphocytes have been found to be closely related to one another, and immune profiling might become a crucial tool in guiding treatment choice.

Despite some limitations, including the limited sample size, long follow-up, and data concerning LDH as an indicator of tumour burden, the study brings some important knowledge to the field, showing that there are immunological effects correlated to the use of CDK4/6 inhibitor as a single agent. Furthermore, to overcome the aforementioned limitations, these results must be investigated in large-scale studies with extensive follow-up and assessment of other inflammatory markers.

## Figures and Tables

**Table 1:** Patients’ characteristics.

Variable	n= 23 Patients
**Age**	
Median	62.8
**Menopause status**	
Pre	5 (22%)
Post	18 (78%)
**Surgery (initial treatment), n (%)**	
Conservative	11 (47.8%)
Mastectomy	12 (52.2%)
**Stage, n (%)**	
IV	23 (100%)
**Metastatic site**	
Brain	16 (70%)
Liver	8 (35%)
Lung	5 (22%)
Node	2 (7%)
**Molecular subtype (primitive tumour), n (%)**	
Luminal A (HR+/HER2−)	7 (30.4%)
Luminal B (HR+/HER2−)	16 (69.6%)
**Adjuvant therapy**	
AI	16 (70%)
ECP + AI	3 (13%)
ECP + LHRH + TAM	4 (17%)
**Type of treatment, n (%)**	
Abemaciclib	8 (34.8%)
Palbociclib	6 (26.1%)
Ribociclib	9 (39.1%)
**Status, n (%)**	
Alive	14 (60.9%)
Dead	9 (39.1%)
**Follow-up (years)**	
Median (IQR)	9.5 (4.8–14.7)

Abbreviations: SD, standard deviation; HR, hormonal receptor; HER2, human epidermal growth factor receptor−2; AI, aromatase inhibitors; ECP, epirubicin + cyclophosphamide (4 cycles) + paclitaxel (weekly); LHRH, gonadotropin-releasing hormone; TAM, tamoxifen; IQR, interquartile range.

**Table 2:** Comparison of values between baseline and after 1 month of therapy.

Variable	Baseline	1 Month	*p*-Value
	(Cells/μL)	(Cells/μL)	
**Lymphocytes abs (CD45+)**			
Mean (SD)	1664.7 (589.2)	1552.4 (701.4)	0.33
**Lymphocytes B (CD19+) abs**			
Median (min–max)	198 (21–484)	125 (8–448)	0.21
**Lymphocytes NK (CD16+CD56+)**			
Mean (SD)	311.6 (152.4)	280.7 (151.3)	0.22
**Lymphocytes T (CD3+) abs**			
Mean (SD)	1134.3 (486.8)	1076.3 (555.1)	0.48
**Lymphocytes T Helper (CD3+CD4+) abs**			
Mean (SD)	680.0 (273.1)	654.2 (314.8)	0.54
**Lymphocytes T Suppressor (CD3+CD8+) abs**			
Mean (SD)	430.7 (233.1)	396.6 (235.1)	0.35
**Lymphocytes Treg (CD3+CD4+CD25++CD127−)**			
Median (min–max)	59 (30–126)	46 (0.0–127)	0.02
**Neutrophils**			
Median (min–max)	3.45 (1.20–9.91)	1.33 (0.71–2.49)	<0.001
**Platelets**			
Mean (SD)	250.8 (67.2)	228.7 (71.8)	0.16
**CD4/CD8 ratio**			
Median (min–max)	1.74 (0.95–4.36)	1.56 (0.69–4.43)	0.55
**N/L ratio**			
Median (min–max)	2.23 (0.77–7.18)	1.00 (0.27–6.00)	<0.001
**P/L ratio**			
Mean (SD)	167.6 (62.9)	1784.2 (87.9)	0.37

Abbreviations: abs, absolute value; SD, standard deviation; N/L, neutrophil to lymphocyte; P/L, platelet to lymphocyte.

**Table 3:** Comparison of values between baseline and after 1 month of therapy according to the type of therapy.

	ABEMACICLIB (n = 8)			PALBOCICLIB (n = 6)			RIBOCICLIB (n = 9)		
Variable	Baseline (Cells/μL)	1 Month (Cells/μL)	*p*-Value	Baseline (Cells/μL)	1 Month (Cells/μL)	*p*-Value	Baseline (Cells/μL)	1 Month (Cells/μL)	*p*-Value
**Lymphocytes Treg (CD3+CD4+CD25++CD127−)** Median (min–max)	58 (36–120)	53 (22–127)	0.53	59 (45–97)	44 (37–84)	0.04	59 (30–126)	45 (0–102)	0.109
**Neutrophils** Median (min–max)	3.32 (2.02–5.78)	1.32 (0.78–2.37)	0.02	3.31 (1.93–9.91)	1.46 (0.71–2.12)	0.03	3.45 (1.20–6.25)	1.39 (0.84–2.49)	0.008
**N/L RATIO** Median (min–max)	2.07 (1.50–4.86)	0.84 (0.46–2.00)	0.02	2.48 (1.79–7.18)	1.11 (0.71–2.16)	0.03	1.75 (0.77–3.88)	0.92 (0.27–6.00)	0.16
**Platelets** Mean (SD)	224.4 (48.8)	187.8 (33.2)	0.03	234.8 (58.7)	232.7 (87.0)	0.96	284.9 (77.39)	262.6 (74.1)	0.44

Abbreviations: SD, standard deviation; N/L, neutrophil to lymphocyte.

**Table 4:** Comparison of SUV variation between baseline and after 1 month of therapy.

Lesions (n= 24)	Baseline	1 Month	*p*-Value
**SUV**			
Median (min–max)	4.8 (1.9–13.0)	2.9 (0.0 −9.9)	0.006
**Lesions Treated with Ribociclib (n = 14)**			
**SUV**			
Median (min–max)	4.75 (3.50–7.25)	2.90 (0.45–5.7)	0.008
**Lesions Treated with Abemaciclib (n = 10)**			
**SUV**			
Median (min–max)	4.85 (2.53–5.93)	3.65 (1.95–6.4)	0.51

## Data Availability

The original contributions presented in the study are included in the article. Further inquiries can be directed to the corresponding authors.
